# Ergothioneine Improves Seed Yield and Flower Number through *FLOWERING LOCUS T* Gene Expression in *Arabidopsis thaliana*

**DOI:** 10.3390/plants13172487

**Published:** 2024-09-05

**Authors:** Tatsuyuki Koshiyama, Yukihiro Higashiyama, Izumi Mochizuki, Tetsuya Yamada, Motoki Kanekatsu

**Affiliations:** 1 New Business Division, Kureha Corporation, Chuo-ku, Tokyo 103-8552, Japan; t-koshiyama@kureha.co.jp (T.K.); higashiyama@kureha.co.jp (Y.H.); 2 United Graduate School of Agricultural Science, Tokyo University of Agriculture and Technology, Fuchu, Tokyo 183-8509, Japan; s244817y@st.go.tuat.ac.jp (I.M.); teyamada@cc.tuat.ac.jp (T.Y.)

**Keywords:** ergothioneine, amino acid, biostimulant, sustainable agriculture, yield, flower number, *FLOWERING LOCUS T*

## Abstract

Biostimulants are a new category of materials that improve crop productivity by maximizing their natural abilities. Out of these biostimulants, those that increase seed production are considered to be particularly important as they contribute directly to the increase in the yield of cereals and legumes. Ergothioneine (EGT) is a natural, non-protein amino acid with antioxidant effects that is used in pharmaceuticals, cosmetics, and foods. However, EGT has not been used in agriculture. This study investigated the effect of EGT on seed productivity in *Arabidopsis thaliana*. Compared with an untreated control, the application of EGT increased the seed yield by 66%. However, EGT had no effect on seed yield when applied during or after bolting and did not promote the growth of vegetative organs. On the other hand, both the number of flowers and the transcript levels of *FLOWERING LOCUS T* (*FT*), a key gene involved in flowering, were increased significantly by the application of EGT. The results suggest that EGT improves seed productivity by increasing flower number through the physiological effects of the FT protein. Furthermore, the beneficial effect of EGT on flower number is expected to make it a potentially useful biostimulant not only in crops where seeds are harvested, but also in horticultural crops such as ornamental flowering plants, fruits, vegetables.

## 1. Introduction

The global population and corresponding food demand are increasing continuously. Consequently, improving crop yields in order to sustain this increase in the global population is essential. Conventional methods for improving crop yield have mainly relied on improvements in plant breeding and the application of fertilizer and chemical pesticides. However, in recent decades, there has been an increase in the demand for environmentally friendly methods of improving yield in sustainable agriculture. Of these new methods, the use of biostimulants has received increasing attention worldwide [1,2,3]. Biostimulants are substances or microorganisms that improve crop yield by maximizing a crop’s potential, such as by improving abiotic stress tolerance and nutrient use efficiency [4]. Research on biostimulants is a growing field, and the size of the global market is increasing [5]. While various effects of biostimulants on crops have been reported, the improvement of seed productivity is of particular importance as it directly affects the yield of cereals such as rice, wheat, barley, and corn, as well as legumes such as soybean and pulses. In addition, since seed formation is associated with fruit development and flower induction, biostimulants that improve seed productivity can also be expected to contribute positively to the yield of flowering plants and fruits.

Amino acid-based biostimulants are categorized into protein amino acids and non-protein amino acids. The effects of protein amino acids on plants have been extensively investigated, and protein amino acid-based biostimulants have been marketed commercially [5,6]. For example, Lardos et al. (2023) reported the effects of using 20 protein amino acids as biostimulants for the growth of *Arabidopsis thaliana* [7]. Their findings showed that glutamine and asparagine promoted plant growth at low concentrations (0.25 mM). On the other hand, although the reports are limited, some non-protein amino acids have been shown to be potentially useful biostimulants. Glycine betaine (N,N,N-trimethyl glycine) is widely known as a non-protein amino acid-based biostimulant and an osmotic regulator that is produced and accumulated in plant cells under conditions of stress [8,9,10]. In addition, the exogenous application of glycine betaine has been shown to improve abiotic stress tolerance in plants [9]. As there are hundreds of non-protein amino acids, it can be expected for there to be others, besides glycine betaine, that are excellent biostimulants for crops.

Ergothioneine (EGT), a naturally occurring non-protein amino acid containing a betaine structure and a thiol group bound to the imidazole ring, is structurally similar to glycine betaine [11,12]. EGT is synthesized by mushrooms and microorganisms, and the thiol–thione tautomerism of EGT makes it well suited for use as a stable antioxidant [12,13,14]. Furthermore, since a specific transporter of EGT has been discovered in human cells, EGT has attracted attention in the food, pharmaceutical, and cosmetics industries [11,15]. While EGT is not synthesized by plants, it has been reported that EGT synthesized by microorganisms is accumulated in plants [11,16,17]. These findings suggest that EGT may perform useful functions as an antioxidant in plants. However, few studies have been reported on the effect of the exogenous application of EGT on plants, EGT has not been commercialized in agriculture, and the effect of EGT as a biostimulant remains to be clarified.

Therefore, in this study, we investigated the effects of EGT as a biostimulant, with a particular focus on seed yield in the model plant *Arabidopsis thaliana*, which is well suited for analyzing the mode of action of biostimulants. Additionally, the effects of EGT on vegetative organs, flower number, and the transcription level of the key flowering gene *FLOWERING LOCUS T* (*FT*) were analyzed, as seed yield is closely related to the growth of vegetative organs such as leaves and roots, as well as the number of flowers.

## 2. Results

### 2.1. Effect of EGT on Seed Yield of A. thaliana

**Figure 1:**
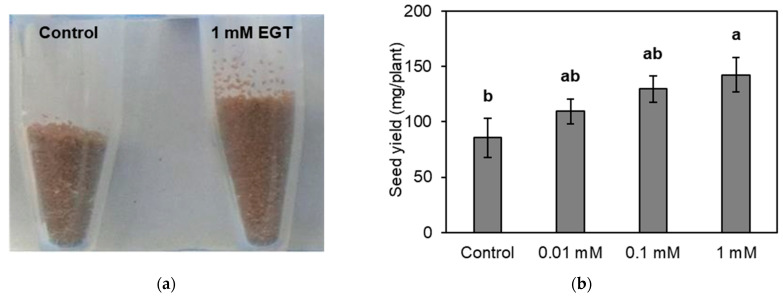
Effects of ergothioneine (EGT) on the seed yield of *Arabidopsis thaliana*: (**a**) seeds harvested at maturity and (**b**) total seed yield per plant. EGT was applied from 3 to 4 weeks after sowing. The values for seed yield are the means of six plants ± SE. Different letters above the bars indicate significant differences according to Tukey’s HSD test (*p* < 0.05).

Seed formation in *A. thaliana* occurs after floral transition, bolting, and pollination, with floral transition being one of the most important stages for maximizing seed yield. Since floral transition and bolting occurred between approximately 5 weeks and 6 weeks after sowing in this study, EGT was applied before floral transition to investigate its effect on seed yield. EGT solution at 0 (control), 0.01, 0.1, and 1 mM was supplied to plants four times every other day from 3 to 4 weeks after sowing, and the seed yield was measured 12 weeks after sowing. The results showed that seed yield increased dose-dependently in response to the application of EGT, and that EGT at 1 mM significantly increased the seed yield by 66% compared to the untreated control (Figure 1). This finding showed that EGT has a beneficial effect on seed yield in *A. thaliana*.

### 2.2. Growth of A. thaliana at the Reproductive Growth Stage

**Figure 2:**
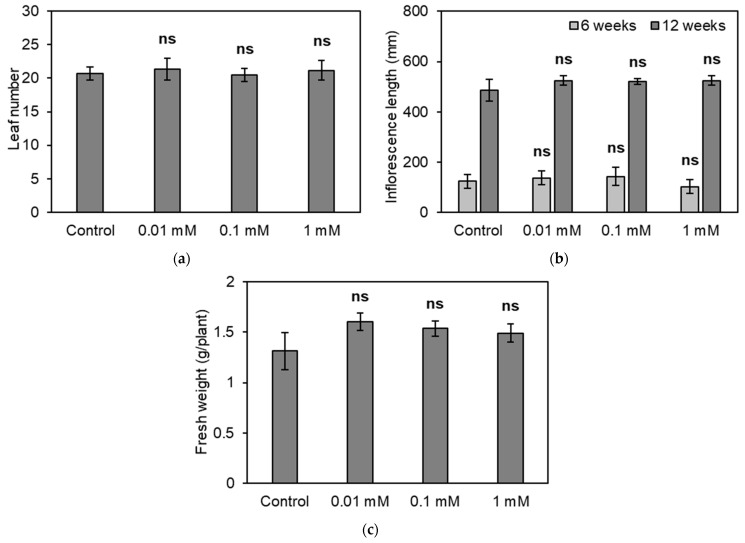
Effects of EGT on plant growth at the reproductive growth stage: (**a**) number of rosette leaves per plant 6 weeks after sowing; (**b**) length of inflorescences 6 and 12 weeks after sowing; and (**c**) fresh weight of aerial parts of the plant 12 weeks after sowing. EGT was applied from 3 to 4 weeks after sowing. All the values are the means of six plants ± SE. “ns” indicates no significant difference from the control, as estimated by Student’s *t*-test (*p* > 0.05).

As shown in Figure 1, the application of EGT prior to bolting increased the seed yield. One possible reason for this effect is that EGT promoted the growth of leaves, which are photosynthetic organs, resulting in increasing seed yields. Therefore, we determined the effect of EGT applied at the same time as that shown in Figure 1 on the growth of aerial parts of the plant at the reproductive growth stage. EGT at concentrations of 0 (control), 0.01, 0.1, and 1 mM was applied four times every other day from 3 to 4 weeks after sowing, and the rosette leaf number, inflorescence length, and weight of the aerial parts of *A. thaliana* were measured. None of the EGT treatments showed a significant difference in the rosette leaf number at 6 weeks, inflorescence length at 6 and 12 weeks, or weight of the aerial parts of *A. thaliana* 12 weeks after sowing (Figure 2). These results suggests that EGT does not increase the size of the whole plant because the inflorescence length and the weight of the aerial parts at harvest did not increase in response to the application of EGT.

### 2.3. Effect of EGT Application at the Reproductive Growth Stage on Seed Yield

**Figure 3:**
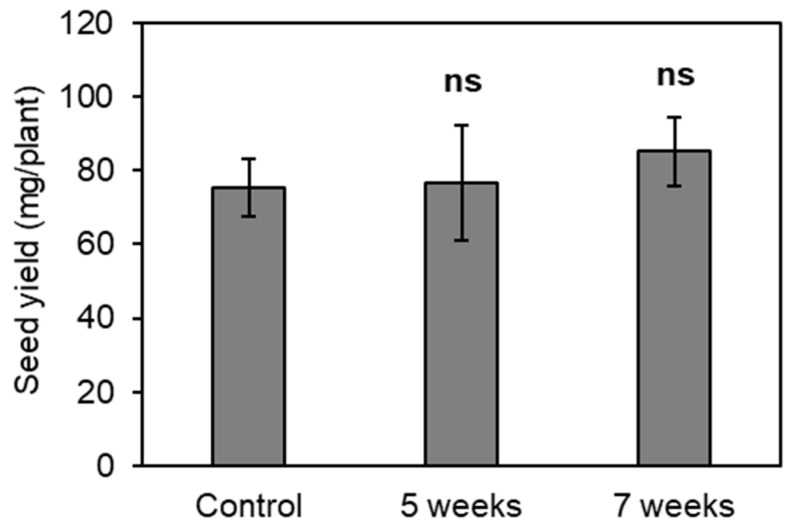
Effects of EGT on the total seed yield. EGT was applied from 5 to 6 weeks after sowing (5 weeks) or from 7 to 8 weeks after sowing (7 weeks). The values of the seed yield are the means of six plants ± SE. “ns” indicates no significant difference from the control, as estimated by Student’s *t*-test (*p* > 0.05).

The results shown in Figure 1 and Figure 2 suggest that EGT influences seed productivity for *A. thaliana*. Therefore, the effect of EGT applied closer to seed formation was investigated. Plants were supplied with 1 mM of EGT four times every other day from 5 to 6 weeks after sowing, during bolting, or from 7 to 8 weeks after sowing, which is after bolting, and the seed yield was measured. The results showed that the application of EGT during or after bolting did not increase the seed yield in *A. thaliana* (Figure 3). Therefore, the application of EGT before floral transition is important for increasing the seed yield, and EGT may influence floral transition in *A. thaliana*.

### 2.4. Effects of EGT on Vegetative Organs of A. thaliana at the Vegetative Growth Stage

**Figure 4:**
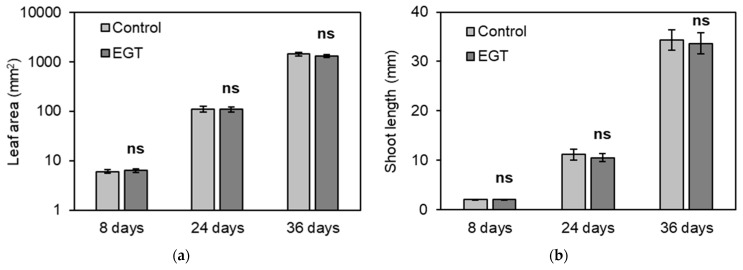

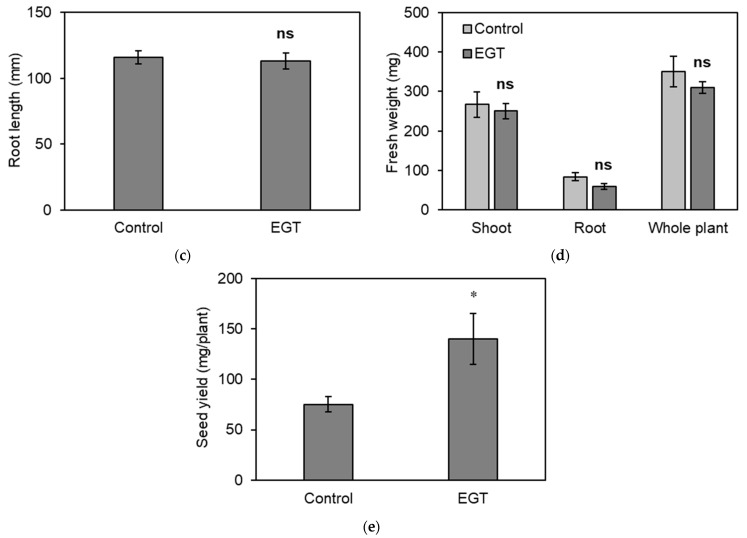
Effects of EGT applied from 1 to 2 weeks after sowing on the growth parameters of seedlings and seed yield. (**a**) Leaf area 1, 3, and 5 weeks after sowing. (**b**) Shoot length 1, 3, and 5 weeks after sowing. (**c**) Root length 5 weeks after sowing. (**d**) Fresh weight of shoots, roots, and whole plants 5 weeks after sowing. (**e**) Total seed yield per plant. All the values are the means of six plants ± SE. The asterisk indicates a significant difference from the control, and “ns” indicates no significant difference from the control, as estimated by Student’s *t*-test (* *p* < 0.05).

EGT applied at the vegetative growth stage increases the seed yield for *A. thaliana*. The application of EGT from 3 to 4 weeks after sowing does not influence the inflorescence length or aboveground mass at harvest. However, it is possible that the timing of EGT application in our study was too late to assess the effect of EGT application on vegetative organs at the vegetative growth stage. To clarify the effects of EGT on the vegetative organs of *A. thaliana* at the vegetative growth stage, seedlings of *A. thaliana* with leaves of the same area and shoots of the same length 1 week after sowing were selected. EGT at a concentration of 1 mM was then applied three times every other day from 1 to 2 weeks after sowing, and growth parameters such as leaf area, shoot length, root length, shoot weight, root weight, and whole plant weight were measured at the vegetative growth stage. Figure 4a–d show that EGT had no significant effect on the leaf area or shoot length 3 weeks after sowing or on leaf area, shoot length, root length, shoot weight, root weight, or whole-plant weight 5 weeks after sowing. To investigate the effect on seed yield at the same time as EGT was supplied, i.e., at the time shown in Figure 4a–d, 1 mM of EGT was applied from 1 to 2 weeks after sowing, and the seed yield was measured. Figure 4e shows that the application of EGT from 1 to 2 weeks after sowing increased the seed yield by 86%. These results suggest that EGT has no beneficial effects on the vegetative organs of *A. thaliana* and that the effect of EGT on the increase in seed yield was not due to the promotion of vegetative organ growth.

### 2.5. Effect of EGT on Flower Number

**Figure 5:**
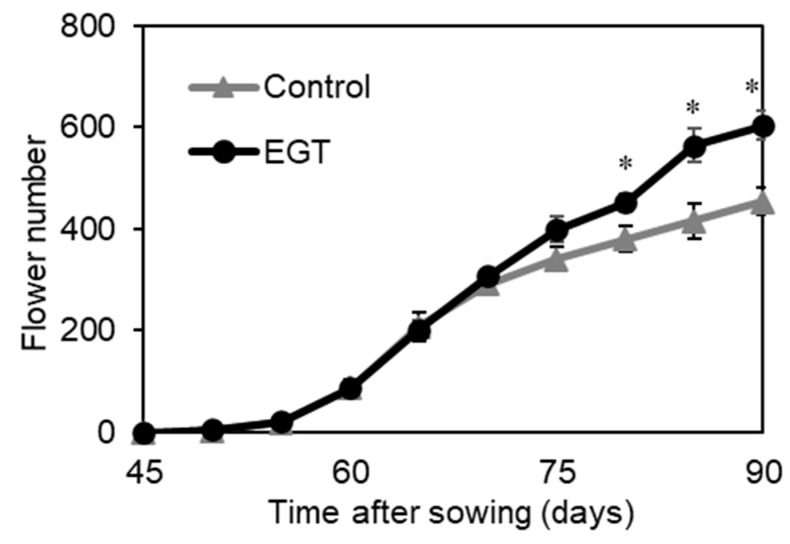
Effects of EGT on the number of flowers from bolting to harvest. EGT was applied from 1 to 2 weeks after sowing. The values for the flower number are the means of six plants ± SE. The asterisks indicate significant differences from the control, as estimated by Student’s *t*-test (* *p* < 0.05).

The application of EGT before floral transition increased the seed yield; however, EGT did not promote the growth of vegetative organs. It was therefore suggested that an increase in flower number may contribute to improving the seed yield. To investigate the effect of EGT on flower number, 1 mM of EGT was applied from 1 to 2 weeks after sowing. To eliminate the effect of the floral transition’s timing on the number of flowers, 6 plants that had started bolting 6 weeks after sowing were selected from 36 plants, and the number of flowers per plant from bolting to harvest was counted. Figure 5 shows that the plants that were treated with EGT had significantly more flowers per plant than the untreated plants 11 weeks after sowing, indicating that the EGT treatment increased the total number of flowers by 33% compared to the untreated control at 13 weeks after sowing. The results show that the increase in the flower number for plants treated with EGT increased the seed number and improved the seed productivity.

### 2.6. Effect of EGT on Gene Expression of Flowering Hormone

**Figure 6:**
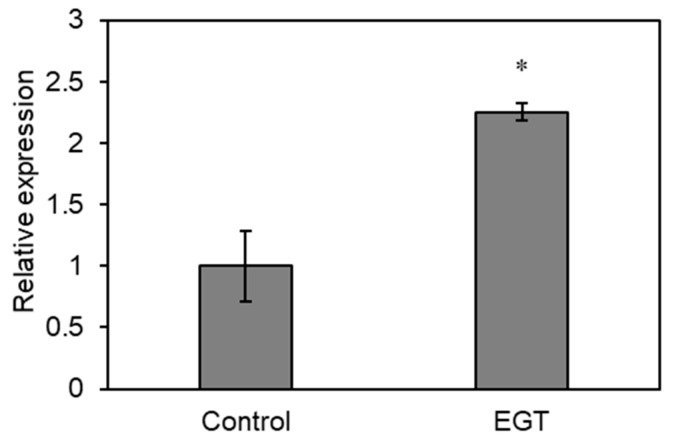
Effects of EGT on the *FLOWERING LOCUS T* expression level in shoots of *A. thaliana* 24 h after application. EGT was applied 1 week after sowing. The values of the relative expression are the means of three biological replicates ± SE. The asterisk indicates a significant difference from the control in Student’s *t*-test (* *p* < 0.05).

Figure 5 shows that EGT increased the flower number, which, in turn, improved the seed yield. The gene product of *FLOWERING LOCUS T* (*FT*) is a flowering hormone that is involved in the induction of floral transition in *A. thaliana*. To clarify whether EGT influences the gene expression of *FT* in *A. thaliana*, RT-qPCR analysis was carried out. EGT at a concentration of 1 mM was applied once one week after sowing, and the level of *FT* transcripts in the shoots of *A. thaliana* was analyzed 24 h after application. Figure 6 shows that EGT increased the relative expression of *FT* by 2.3 times compared to the untreated control. This result suggests that EGT promotes the floral transition of *A. thaliana* through the physiological effects of the FT protein, resulting in an improvement in flower number and seed productivity.

## 3. Discussion

EGT, a natural amino acid which is structurally analogous to glycine betaine, has recently gained attention for its possible use as a biostimulant. Nonetheless, relatively few studies have been conducted to clarify the beneficial effects of EGT in this regard. Our findings showed that EGT has a positive effect on seed yield in the model plant *A. thaliana* if applied before bolting. In addition, (i) the effect of EGT on seed yield was not observed if EGT was applied during or after bolting, (ii) EGT did not promote the growth of vegetative organs such as leaves, shoots, and roots, and (iii) the number of flowers and the transcript level of the key flowering gene, *FT*, increased significantly when EGT was applied one week after sowing. These results strongly suggest that EGT is effective as a biostimulant and that it improves seed productivity by increasing the flower number through the physiological action of the FT protein acting as a flowering hormone. While various biostimulants have been shown to increase the seed yield by improving vegetative organs [3], studies on the research and development of biostimulants for improving seed productivity through flower induction are limited. The beneficial effects of EGT on flower number and seed yield are expected to make it a potentially useful biostimulant, not only in crops where seeds are harvested, but also in horticultural crops such as ornamental flowering plants, fruits, and vegetables.

Although EGT was not observed to promote the growth of vegetative organs in this study, similar results have been reported using sclerothionene (2-hydroxyethyl-ergothioneine), which is an analog of EGT. For example, Matsuo and Satomura (1968) showed that sclerothionene promoted the growth of shoots and roots in rice seedlings, while EGT had no effect on vegetative organs [18]. However, this research did not examine the effects of EGT on flower number and seed yield in plants. In this study, for the first time, we found that EGT has a positive effect on flower number and seed yield. These results show that EGT does not increase the size of the whole plant body or vegetative organs, but it does promote increases in the number of flowers and seed production.

In comparisons to EGT application before and after floral transition, application before floral transition increased seed production, whereas application after floral transition did not increase seed production. These results suggest that the application of EGT influences floral transition. Furthermore, by investigating the transcriptional levels of *FT* in *A. thaliana*, we found that EGT increased the expression of the *FT* gene. In *Arabidopsis*, the photoperiod, ambient temperature, age, gibberellins, vernalization, and autonomous pathway are all known to affect the regulation of *FT* transcription. The FT protein is produced in the leaves and transported to the shoot apical meristem (SAM) through the phloem. At the SAM, the FT protein interacts with transcription factor FD via the 14-3-3 protein to initiate transition to the reproductive phase [19,20]. Detailed analysis of the relationship between these factors and EGT is considered important for elucidating the mode of action of EGT in floral transition. In tomato, *SINGLE FLOWER TRUSS* (*SFT*), which is an ortholog of *FT* in *A. thaliana*, has been reported to influence fruit yield [21]. Moreover, a study on the use of brown seaweed extracts as biostimulants showed that the upregulation of *SFT* through the application of brown seaweed extracts to tomato indirectly improved plant flowering phenotypes, such as by increasing the number of flowering buds, in the hypothesized mechanism [22]. Therefore, the findings of this study suggest that the application of EGT induces an increase in flower numbers in *A. thaliana* through increasing the transcription levels of *FT*.

While the mode of action of the effect of EGT on the transcription of *FT* remains unclear, studies on biostimulants similar to EGT have been reported. For example, proline, an amino acid-based biostimulant which is similar to EGT, is a well-known osmotic regulator that stimulates antioxidant systems in plant. Its exogenous application improves abiotic stress tolerance [9,23]. In addition, it has been reported that proline affects the flowering time and expression of *FLOWERING LOCUS C*, which is important in floral transition in *Arabidopsis* [24]. Glutathione, a tripeptide with a thiol group similar to ergothioneine, is one of the most important antioxidants that respond to environmental stress in plants. Its exogenous application also increases abiotic stress tolerance [25]. In addition, glutathione is also known to affect flowering in *Arabidopsis* [26]. Another antioxidant, ascorbic acid, has also been proposed to affect flowering in plants [27]. Reactive oxygen species are suggested to act as inducers of flowering in plants [28]. It is therefore possible that the effects of proline, glutathione, and ascorbic acid on antioxidant system in plants may influence flowering. Since antioxidant EGT is known to protect cells from oxidative stress, further studies may help elucidate the mode of action of EGT and how it increases the transcription levels of *FT*.

Increasing seed production via EGT is extremely important for cereals and legumes, which play very important roles in global food production. In addition, since the increase in flower number leads to an increase in fruit number, the effect of EGT on increasing flower number observed in this study is important not only for crops from which seeds are harvested, but also for crops from which fruits and flowers are harvested. A limited number of studies have reported the effect of biostimulants in terms of increasing flower numbers in fruits and vegetables. For example, seaweed extracts have been shown to have a positive effect on flower numbers and fruit sets in eggplants [29]. This study shows the effect on only one model species in a controlled experimental system. Therefore, in future studies, the effects of EGT in crops from which fruits are harvested, such as tomato and strawberry, should be investigated under field conditions. The findings of this study provided basic data that will promote the use of ergothioneine in agricultural applications. Further studies using *Arabidopsis* and other plants are needed to understand the mode of action of EGT in increasing flower number and seed yield.

## 4. Materials and Methods

### 4.1. Plant Material and Growth Conditions

Seeds of *Arabidopsis thaliana* (ecotype Columbia-0) were purchased from Funakoshi Co., Ltd., Tokyo, Japan. The plants were grown in 60 mm diameter pots (one plant per pot) containing granular soil (Kumiai horticultural soil contained 340, 1350, 220, and 150 mg/L of N, P, K, and Mg, respectively) and vermiculite mixture (3:1, *v*/*v*) in a growth chamber MLR-352H-PJ (PHC Corporation, Tokyo, Japan) at 22 °C and 55% relative humidity, with a photon flux density of photosynthetically active radiation of approximately 60 μmol m^−2^ s^−1^ under long-day conditions (16 h light and 8 h dark). All plant seeds were harvested at maturity and weighed to determine the seed yield.

### 4.2. Application Conditions

L-ergothioneine (EGT) was purchased from Funakoshi Co., Ltd., Tokyo, Japan. The compound was dissolved in water. Instead of supplying the plants with water, 50 mL of a 0.01, 0.1, or 1 mM EGT solution was added to separate saucers placed under six plant pots (approximately 0.019, 0.19, or 1.9 mg of EGT per plant, respectively) every other day.

### 4.3. Growth Parameters

The length and width of all *A. thaliana* leaves were measured with a caliper to calculate the leaf area. The length of the shoots, roots, and inflorescences of *A. thaliana* was measured with a ruler. The fresh weight was determined immediately after harvesting the plants.

### 4.4. RNA Extraction and cDNA Synthesiss

Total RNA for RT-qPCR was extracted from the shoots of one-week-old seedlings using an ISOSPIN Plant RNA kit (NIPPON GENE Co., Ltd., Tokyo, Japan), according to the manufacturer’s instructions. Each biological replicate comprised a pool of two plants. The purity and concentration of RNA in the extracts were determined using a NanoDrop spectrophotometer (Thermo Fisher Scientific, Inc., Waltham, MA, USA) and a Qubit 2.0 fluorometer (Thermo Fisher Scientific, Inc., Waltham, MA, USA). cDNA synthesis was performed using a PrimeScript RT reagent Kit with gDNA Eraser (Takara Bio Inc., Shiga, Japan), following the manufacturer’s instructions. The cDNA was stored at −20 °C until analysis.

### 4.5. RT-qPCR Analysis

A mixture containing 5 µL of KAPA SYBR FAST qPCR Kit Master Mix (2×) Universal (Kapa Biosystems, Inc., Wilmington, MA, USA), 0.2 µL of 10 µM Forward primer, 0.2 µL of 10 µM Reverse primer, 3.6 µL of PCR-grade water, and 1 µL of cDNA solution was added to each well of a 48-well PCR plate. The PCR reaction solution was subjected to initial denaturation at 95 °C for 10 min followed by 40 cycles of denaturation at 94 °C for 10 s and annealing and extension at 60 °C for 30 s, using an Eco Real-Time PCR System (Illumina, Inc., San Diego, CA, USA). Finally, melting curve analysis was performed from 60 °C to 95 °C, and the plate was incubated at 4 °C. The mRNA copy number for each gene in 10 ng of total RNA of each sample was determined by comparison to a standard curve. The mRNA copy number for *UBC21*, a housekeeping gene, was used as an internal standard and for normalization to determine the relative transcript amounts. RT-qPCR analysis was performed in triplicate. The primers used in this study are listed in Table 1.

### 4.6. Statistical Analysis

All experiments were repeated three or six times. The data are expressed as the mean with the standard error (SE). *p*-values of 0.05 or less were considered to be statistically significant. Tukey’s HSD test and Student’s *t*-test were used to determine significant differences from the control in this study. All statistical analyses were performed with EZR [30], which is a graphical user interface for R (The R Foundation for Statistical Computing, Vienna, Austria). More precisely, it is a modified version of R commander designed to add statistical functions frequently used in biostatistics.

## Figures and Tables

**Table 1:** List of primers used in this study.

Gene Name	Locus	Forward Primer (5′ to 3′)	Reverse Primer (5′ to 3′)
*FLOWERING LOCUS T*	AT1G65480	CTACAACTGGAACAACCTTTGGC	CGAGTGTTGAAGTTCTGGCG
*UBC21*	AT5G25760	TCCTCTTAACTGCGACTCAGG	GCGAGGCGTGTATACATTTG

## Data Availability

The data that support the findings of this study are available from the corresponding author upon reasonable request.
